# Corticosteroids alleviate lipopolysaccharide‐induced inflammation and lung injury via inhibiting NLRP3‐inflammasome activation

**DOI:** 10.1111/jcmm.15849

**Published:** 2020-09-25

**Authors:** Jia‐Wei Yang, Bei Mao, Ru‐Jia Tao, Li‐Chao Fan, Hai‐Wen Lu, Bao‐Xue Ge, Jin‐Fu Xu

**Affiliations:** ^1^ Department of Respiratory and Critical Care Medicine Shanghai Pulmonary Hospital Tongji University School of Medicine Shanghai China; ^2^ Department of Shanghai Key Laboratory of Infectious Diseases Shanghai Pulmonary Hospital Tongji University School of Medicine Shanghai China; ^3^ Department of Microbiology and Immunology Tongji University School of Medicine Shanghai China

**Keywords:** acute lung injury, corticosteroids, mitochondrial reactive oxygen species, NLRP3‐inflammasome

## Abstract

The role of corticosteroids in acute lung injury (ALI) remains uncertain. This study aims to determine the underlying mechanisms of corticosteroid treatment for lipopolysaccharide (LPS)‐induced inflammation and ALI. We used corticosteroid treatment for LPS‐induced murine ALI model to investigate the effect of corticosteroid on ALI in vivo. Moreover, LPS‐stimulated macrophages were used to explore the specific anti‐inflammatory effects of corticosteroids on NLRP3‐inflammasome in vitro. We found corticosteroids attenuated LPS‐induced ALI, which manifested in reduction of the alveolar structure destruction, the infiltration of neutrophils and the inflammatory cytokines release of interleukin‐1β (IL‐1β) and interleukin‐18 (IL‐18) in Lung. In vitro, when NLRP3‐inflammasome was knocked out, inflammatory response of caspase‐1 activation and IL‐1β secretion was obviously declined. Further exploration, our results showed that when corticosteroid preprocessed macrophages before LPS primed, it obviously inhibited the activation of caspase‐1 and the maturation of IL‐1β, which depended on inhibiting the nuclear factor‐κB (NF‐κB) signal pathway activation. However, when corticosteroids intervened the LPS‐primed macrophages, it also negatively regulated NLRP3‐inflammasome activation through suppressing mitochondrial reactive oxygen species (mtROS) production. Our results revealed that corticosteroids played a protection role in LPS‐induced inflammation and ALI by suppressing both NF‐κB signal pathway and mtROS‐dependent NLRP3 inflammasome activation.

## 1 INTRODUCTION

The inflammasome is a complex of proteins complexes, which has four known structural subsets including nucleotide‐binding oligomerization domain receptors (NLR) family, pyrin domain‐containing 1 (NLRP1), NLRP3, NLR family CARD domain‐containing protein 4 (NLRC4) and absent in melanoma 2 (AIM2).^1^, ^2^, ^3^ The inflammasome has a basic structure contains the adaptor protein ASC (apoptosis‐associated speck‐like protein containing caspase‐1 activator domain), which recruit and activate pro‐caspase‐1 when triggered by various pathogenic, environmental or endogenous danger signals. It will turn into active caspase‐1, which cleaves the cytokine precursors interleukin‐1β (pro‐IL‐1β) and interleukin‐18 (pro‐IL‐18) to their mature and biologically active pro‐inflammatory factors IL‐1β and IL‐18.^4^, ^5^, ^6^ These inflammasome‐activated cytokines play critical roles in the propagation of the acute inflammatory response.

The NLRP3 inflammasome is most well‐characterized inflammasome of the innate immune system, consisting of nucleotide‐binding‐domain, leucine‐rich repeat domain‐containing protein (NLRP) and an N‐terminal pyrin domain, the adaptor protein ASC,^7^ which express in many inflammatory cells, including macrophages and neutrophils. It is activated by many factors including endogenous danger signals (ATP, uric acid crystal), pathogens (bacteria, viruses, etc), and distinct pathogen associated molecular patterns (PAMPs).^8^, ^9^, ^10^, ^11^ There are two‐step mechanisms for full activation of NLRP3 inflammasome. First, priming signal such as LPS recognizes Toll‐like receptors (TLRs) and induces production of pro‐IL‐1β and pro‐IL‐18. Second, some cellular danger signals converge on a pathway that involving in dysregulating ionic balance such as potassium (K+) efflux, pore formation in cell membranes, lysosome and mitochondrial damage, leading to the release of cathepsins and elevating of reactive oxygen species (ROS), which are responsible for NLRP3 activation. Then, it activates casepase‐1 to cleave pro‐IL‐1β and pro‐IL‐18 to be their active form.^12^, ^13^ It is apparent that TLRs agonism is requirement for activation of NLRP3 inflammasome and each of these factors in NLRP3 inflammasome activation is stimulus dependent.

As shown in previous studies, the NLRP3‐inflammasome and its regulated inflammatory responses such as caspase‐1‐dependent IL‐1β and IL‐18 maturation and secretion are crucial for ALI.^14^, ^15^, ^16^, ^17^, ^18^, ^19^ Moreover, corticosteroids have been considered as a potential therapy for ALI due to their anti‐inflammatory and immunomodulatory effects. In our study, we found that NLRP3‐inflammasone played a critical role in murine ALI and inflammation of murine macrophages, and corticosteroid treatment alleviated ALI in mice. Therefore, we hypothesize whether corticosteroids affect the outcome of ALI via mediating the activation of NLRP3 inflammasome. These investigations promoted us to explore the underlying mechanism of corticosteroid effect on NLRP3‐inflammasome.

## 2 MATERIALS AND METHODS

### 2.1 Murine preparation and in vivo experiments

#### 2.1.1 Animal experiments

Pathogen‐free C57B/L6 and NLRP3−/− mice were housed in a specific pathogen‐free (SPF) environment. Eight male mice (8 weeks old, more or less 20 g) in each group were used for animal experiments. All animal experiments were approved by the Animal Care and Use Committee of Tongji University (2013‐FKA03). Experimental mice received 60 µg LPS (L4391, Sigma‐Aldrich) intratracheally instillation inspiration in a volume of 30 µl phosphate‐buffered saline (PBS) for ALI. Control mice received a volume of 30 µl PBS intratracheally instillation inspiration. Two hours later, mice were intraperitoneally injected with 5 mg/kg dexamethasone (DEX) or equal volume PBS. After 8th, we collected blood, BALF and lung tissue samples of these animals. There are six groups of mice. Control groups: WT/PBS/PBS, WT/PBS/DEX, NLRP3−/−/PBS/PBS. Experimental groups: WT/LPS/PBS, WT/LPS/DEX, NLRP3−/−/LPS/PBS.

Blood samples were collected by extracting the eyeball. After clotted for two hours, we transferred the serum to a mew 1.5 mL microcentrifuge tube stored at −80°C for cytokines detection. Lungs were lavaged with a total volume of 1 mL PBS (pH 7.4), immediately placed on ice and then centrifuged at 376 *g* on a 4°C tabletop centrifuge for 10 minutes. Supernatants of BALF were stored at −80°C for further assays. The cell precipitate was suspended with PBS and then used the cell counter to count the total cell number of each sample after eradicating the red blood cells. Different cellular types were obtained by flow cytometry. Left lungs were harvest in 1 mL PBS and grinded homogenate stored at −80°C for further detection. Right lungs were made into pathological section with haematoxylin‐eosin (H&E) stained, observed at light microscopy, and the lesions were defined as follows: (a) alveolar damage, (b) haemorrhage, (c) infiltration or aggregation of neutrophils and (d) thickness of alveolar wall or hyaline membrane formation.^20^

### 2.2 Cell preparation and stimulation

Thioglycollate‐elicited peritoneal macrophages were generated as previous study.^21^ Unless otherwise indicated, macrophages were primed with 500 ng/mL LPS (L4391, Sigma‐Aldrich) for 4 hours, before treatment with dexamethasone (D4902, Sigma‐Aldrich, at doses and times as indicated in the figure legends), followed by stimulation with 20 µmol/L nigericin (Nig, 4312, tocris) for 15 minutes or 5 mmol/L Adenosine Triphosphate (ATP, A2383, Sigma‐Aldrich) for 30 minutes.

#### 2.2.1 Western blotting

We detected IL‐1β and caspase‐1 cleavage by immunoblotting. Isolated protein from supernatant was precipitated with methanol and chloroform. Briefly, 500 μL supernatant is fully mixed with 500 μL of methanol and 125 μL of chloroform. After centrifuge, a protein layer would be visible. Washed them with 500 ul methanol and dried. Then, boiled protein sample in the 40 ul loading buffer at 95°C for 10 minutes and separated by SDS/PAGE, followed by transfer to polyvinylidene difluoride membranes (PVDF). Immunoblots were incubated with primary antibodies against caspase‐1 (sc‐514, Santa Cruz) and IL‐1β (5129‐100, BioVision). Immunoblots from cell lysates incubated with antibodies against NLRP3 (15 101, Cell Signaling Technology) and NF‐κB (pp65). Proteins were detected by enhanced chemilumiescense.

#### 2.2.2 ELISA for cytokines

The mouse IL‐1β and IL‐18 ELISA kit was from e‐Bioscience. Mouse cytokines in culture supernatants, serum, BALF or lung tissue lyses were measured with ELISA kits according to the manufacturer's protocol.

#### 2.2.3 Cytotoxicity assays

Peritoneal macrophages from WT or NLRP3−/− mice were plated in 6‐well plates stay overnight. At the second day, the medium was replaced with fresh medium before giving the corresponding stimulation as the cell preparation and stimulation shown. Cell membrane damage was determined at the indicated time points by LDH release in supernatant using an LDH detection kit (Promega) according to the manufacturer's instructions.

### 2.3 Flow cytometry

Cells harvested from BALF were incubated with appropriate dilutions of macrophage cell antibody panel (CD11b + F4/80 + Gr1‐/low) or neutrophil cells antibody panel (CD11b + F4/80‐Gr+/high) and then washed. The cells were harvested and resuspended in PBS solution containing 1% FBS for FACS analysis. The data were acquired with a Flow Cytometer (BD AccuriTM) and used FlowJo analytical software (X10) to analyse the related proportion of neutrophil and macrophage cells. Mitochondrial ROS (mtROS) were measured in cells by MitoSOX Red (Invitrogen) staining (5 μmol/L for 10 minutes at 37°C, protected from light). Briefly, cells were stained for 10 minutes at 37°C with 5 μmol/L MitoSox red, followed by 15 minutes of nigericin incubation. Cells were washed with PBS, treated with trypsin and resuspended in PBS containing 1% heat‐inactivated foetal bovine serum (FBS). Data were acquired with a Flow Cytometer (BD AccuriTM) and were analysed with FlowJo analytical software (×10).

### 2.4 Confocal microscopy

Macrophages were plated on coverslips, stimulated as described previous, the MitoTracker Green, MitoSOX Red and DAPI were used to label total mitochondria, mtROS and the nuclei, respectively, for 30 minutes at 37°C, protected from light. Cells were then washed with warm PBS three times. Confocal images were obtained with a Nikon confocal laser scanning microscope.

### 2.5 Statistics analysis

Continuous variables were described by mean ± SD (standard deviation) or median with interquartile range (IQR, 25%‐75%), as appropriate. Categorical variables were described by absolute numbers (percentages). Student's *t* test and Mann‐Whitney U‐test were used to analyse normally and non‐normally distributed continuous data, respectively. Categorical variables were analysed by the chi‐square test. *P* < .05 considered to be significant.

For in vivo and in vitro experiments, each experiment was repeated for at least three time, data are presented as mean ± SD and the Student t test was used to compare differences between groups unless otherwise specified. A two‐tail *P*‐value < .05 was considered statistically significant.

The statistical package SPSS (version 19.0; SPSS, Chicago, IL, USA) was used for statistical analysis and GraphPad Prism (version 5; GraphPad Software, San Diego, CA, USA) was used for drawing graphs.

## 3 RESULTS

### 3.1 NLRP3‐inflammasome is essential for LPS‐induced ALI and corticosteroid treatment alleviate LPS‐induced ALI

**FIGURE 1:**
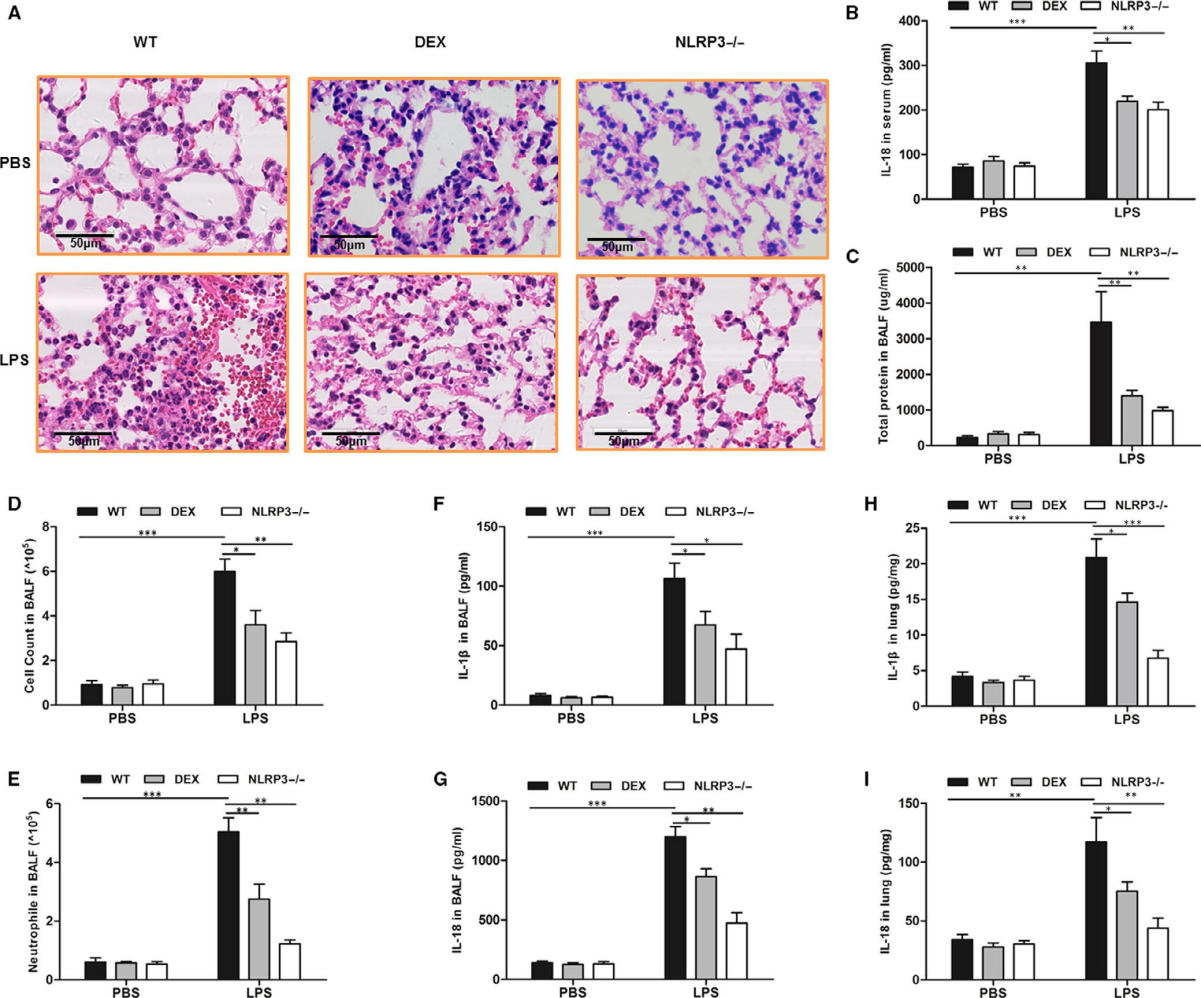
NLRP3‐inflammasome is essential for LPS‐induced ALI and corticosteroid treatment alleviate LPS‐induced ALI. WT and NLRP3−/− mice received LPS intratracheally instillation inspiration for ALI. A, H&E staining of lungs at a magnification of 400× was determined in different models. B, IL‐18 levels in serum in different models. C, Total protein in BALF in different models. D, Total cell count in BALF in different models. E, cell count of neutrophils in BALF in different models. F, IL‐1β levels in BALF in different models. G, IL‐18 levels in BALF in different models. H, Concentrations of IL‐1β in lung homogenates in different models. I, IL‐18 in lung homogenates in different models. *P < .05, **P < .01 and ***P < .001

As we have seen, the absence of NLRP3 significantly attenuated the LPS‐induced model of ALI. Similarly, corticosteroid treatment significantly attenuated LPS‐induced ALI. The reductions of destruction of the alveolar structure and inflammatory cell infiltrations of lung tissue were found in NLRP3‐deficient mice and corticosteroid treatment mice by haematoxylin‐eosin staining (H&E) (Figure 1A). When the NLRP3‐inflammasome was knocked out, the level of IL‐18 in the serum (Figure 1B) and the total protein concentration in BALF (Figure 1C) were also found to be reduction. Corticosteroid treatment had the similar effect. Moreover, the cell counts in BALF decreased significantly (Figure 1D), especially the number of neutrophils (Figure 1E) in NLRP3−/− group and corticosteroid treatment group. Furthermore, the NLRP3−/− mice had the lower levels of IL‐1β and IL‐18 in the BALF during LPS‐induced ALI. Corticosteroid treatment reduced the levels of IL‐1β and IL‐18 in the BALF of LPS‐induced ALI mice (Figure 1F,G). Similarly, the levels of IL‐1β and IL‐18 in the lung homogenate were reduced in NLRP3‐inflammasome knocked out mice and corticosteroid treatment mice (Figure 1H,I).

### 3.2 *NLRP3‐inflammasome mediated acute inflammation* in vitro

**FIGURE 2:**
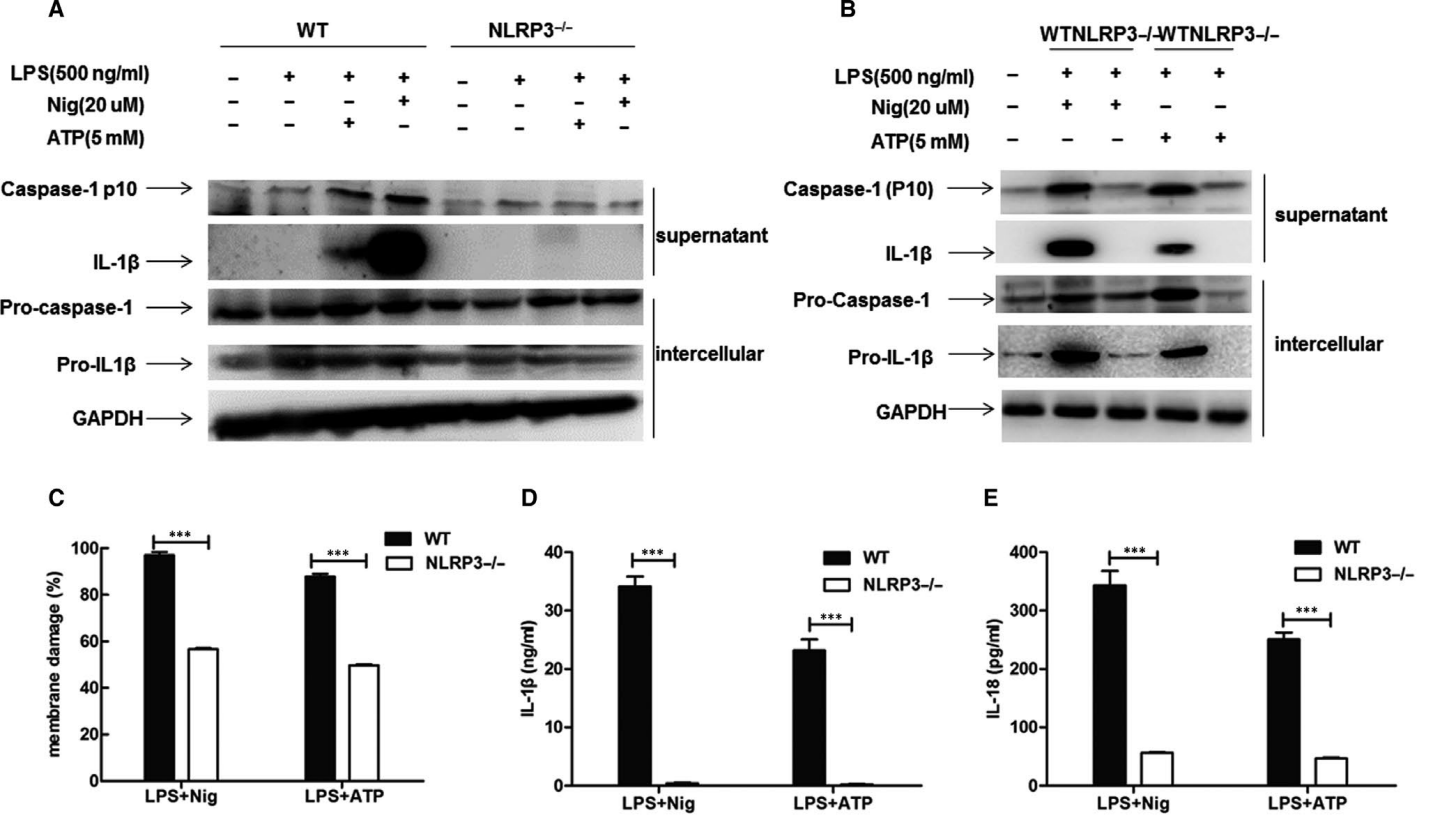
NLRP3‐inflammasome mediated pro‐inflammatory cytokine responses in vitro. Peritoneal macrophages from WT or NLRP3−/− mice were stimulated with LPS (500 ng/mL) for 4 h, followed by incubation with nigericin (Nig) (20 µmol/L) for 15 min or adenosine triphosphate (ATP) (5 mmol/L) for 30 min. A and B, Western blot analysis of caspase‐1 and IL‐1β activation in supernatant. C, Percentages of membrane damage were evaluated by LDH cytotoxicity assay (Promega) in the culture supernatant. D, Concentration of IL‐1β in the supernatant was detected by ELISA. E, Concentration of IL‐18 in the supernatant was detected by ELISA

We isolated murine peritoneal macrophages from wild‐type (WT) and NLRP3‐inflammasome deficient (NLRP3−/−) mice to explore the importance of the NLRP3‐inflammasome in vitro. When WT murine peritoneal macrophages were challenged with LPS and nigericin or ATP, it caused NLRP3‐inflammasome activation, as shown in the expression of mature caspase‐1 and IL‐1β secretion. When NLRP3‐inflammasome was knocked out, the expression of caspase‐1 activation and IL‐1β secretion was almost absent in macrophages in response to pro‐inflammatory stimuli (Figure 2A,B). The cell damage was significantly reduced in NLRP3−/− macrophages relative to WT macrophages by detecting the LDH levels in the culture supernatant (Figure 2C). Similar results were found in the concentrations of IL‐1β and IL‐18 in cell culture supernatants (Figure 2D,E).

### 3.3 Corticosteroid suppressed NLRP3‐inflammasome via inhibiting NF‐κB signal pathway

**FIGURE 3:**
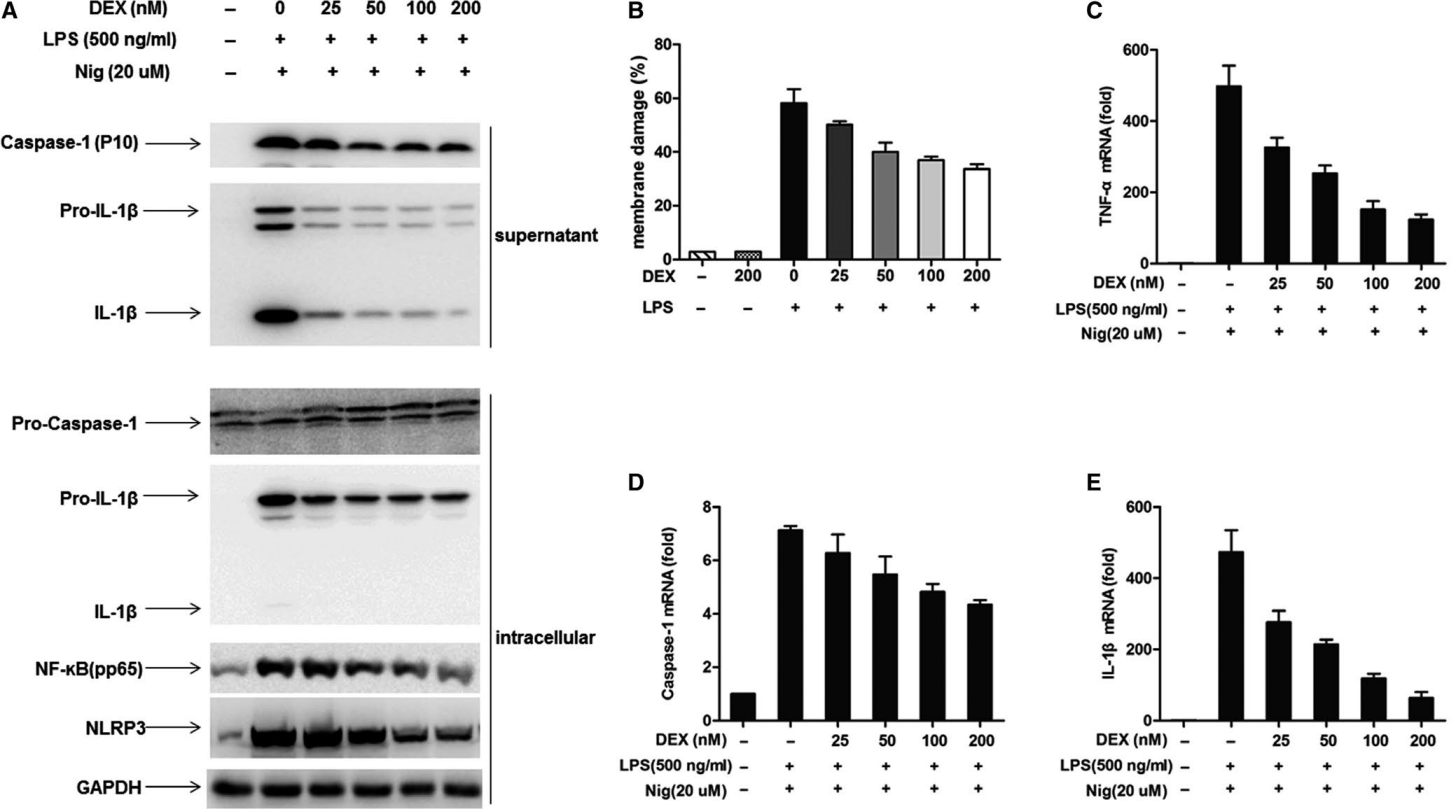
Corticosteroids suppressed NLRP3‐inflammasome activation *via* inhibiting NF‐κB pathway. Peritoneal macrophages from WT mice were treated with various dosages of dexamethasone before challenged with LPS (500 ng/mL) for 4 h and then nigericin (Nig) (20 µmol/L) for 15 min. A, Western blot analysis showed that dexamethasone inhibited caspase‐1, IL‐1β in culture supernatant. The expression of NLRP3‐inflammasome, pro‐caspase‐1, pro‐IL‐1β and NF‐ĸB (p65) also suppressed by dexamethasone, which were in dose‐dependent manner. B, Dexamethasone exerted a protective effect on LPS‐induced cell damage. C, Transcription of TNF‐a was inhibited by various dosages of dexamethasone. D, Transcription of Caspase‐1 was inhibited by various dosages of dexamethasone. E, Transcription of IL‐1β was inhibited by various dosages of dexamethasone

When murine peritoneal macrophages were treated with dexamethasone prior to challenging with LPS and nigericin, dexamethasone significantly inhibited pro‐caspase‐1 and pro‐IL‐1β. Additionally, the expression of mature caspase‐1 and IL‐1β secretion was dose‐dependently inhibited in cell culture supernatants. Likewise, the activation of NF‐κB (pp65) and NLRP3 proteins was markedly suppressed by dexamethasone, in a dose‐dependent manner (Figure 3A). Macrophage activity was not affected by dexamethasone, and it had a protective effect on acute cell injury induced by LPS (Figure 3B). Dexamethasone also inhibited the transcription of tumour necrosis factor‐alpha (TNF‐a), Caspase‐1 and IL‐1β, which were the classical cytokines regulated by the NF‐κB‐dependent signalling pathway (Figure 3C‐E). These results revealed that dexamethasone suppressed the first signal pathway of NLRP3‐inflammasome activation from the transcriptional process by inhibiting the NF‐κB signal pathway.

### 3.4 Corticosteroid inhibited Caspase‐1 activation and IL‐1β release

**FIGURE 4:**
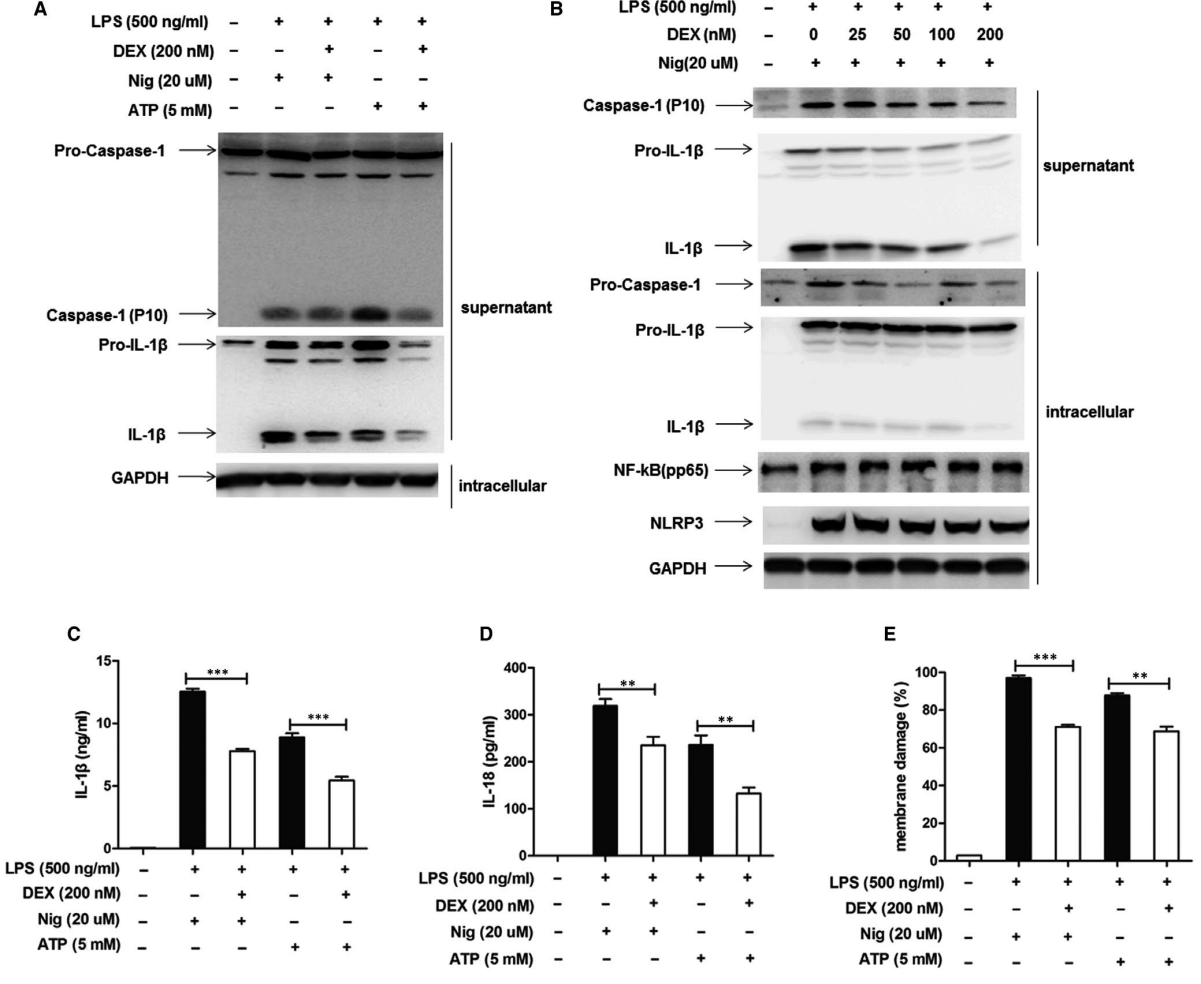
Dexamethasone negatively regulated caspase‐1 activation and IL‐1β secretion. We gave the LPS‐primed murine macrophages dexamethasone treatment stimulated followed by adenosine triphosphate (ATP) (5 mmol/L) for 30 min or nigericin (Nig) (20 µmol/L) for 15 min. A, Western blot analysis showed that dexamethasone inhibited caspase‐1 activation and IL‐1β secretion. B, Western blot analysis showed that dexamethasone inhibited caspase‐1 and IL‐1β activation in dose‐dependent manner. C, Dexamethasone inhibited the concentrations of IL‐1β in supernatant. D, Dexamethasone inhibited the concentrations of IL‐18 in supernatant. E, Percentages of membrane damage were evaluated by LDH cytotoxicity assay (Promega) in the culture supernatant

When murine peritoneal macrophages were stimulated by LPS prior and then received the dexamethasone treatment before ATP or nigericin stimulated, Western blotting revealed that the caspase‐1 activation and IL‐1β secretion in cell culture supernatants were still suppressed by dexamethasone (Figure 4A). In addition, the expression of caspase‐1, IL‐1β and NLRP3 diminished gradually with the increased dose of dexamethasone. The inhibition of dexamethasone on pro‐caspase‐1 and pro‐IL‐1β in intracellular was slight. However, the expression of NF‐κB (pp65) was not affected by dexamethasone (Figure 4B). The concentrations of IL‐1β and IL‐18 in cell culture supernatants decreased after dexamethasone treatment (Figure 4C,D). Dexamethasone exerted protection against cell damage during the acute inflammatory response, which was detected by LDH release in cell culture supernatants (Figure 4E).

### 3.5 Corticosteroid suppressed NLRP3‐inflammasome via inhibiting mtROS production

**FIGURE 5:**
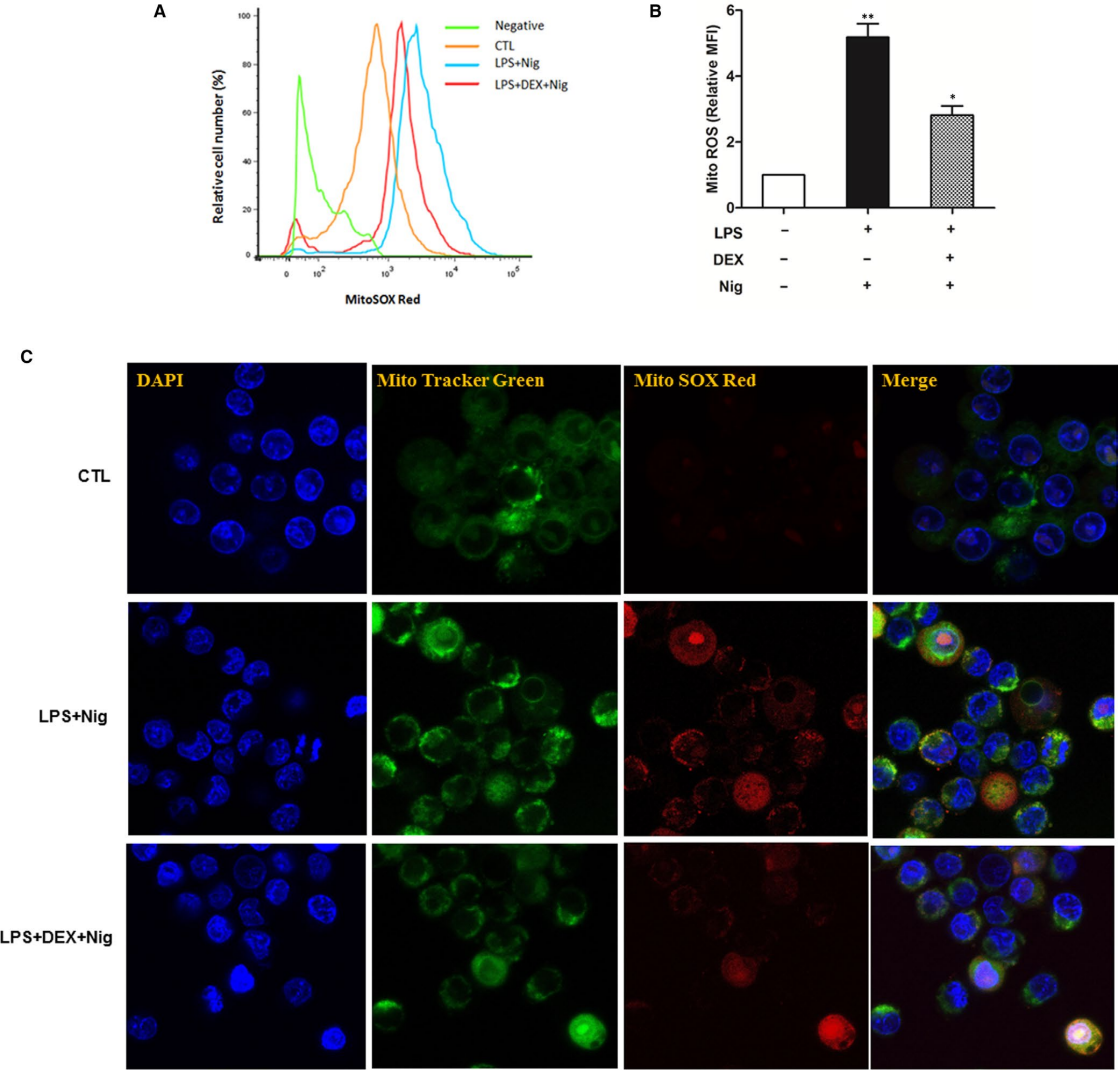
Corticosteroids inhibited mtROS generation. We gave LPS‐primed macrophages dexamethasone treatment stimulated followed by nigericin (Nig) (20 µmol/L) for 15 min. MitoTracker was used to detect mitochondria, MitoSOX Red was used to detect ROS, and DAPI was used to stain the nuclei of the cells. A, Dexamethasone inhibited ROS generation by flow cytometry. B, Dexamethasone inhibited mean fluorescence intensity of ROS by flow cytometry. C, Dexamethasone inhibited ROS generation by confocal microscopy (LPS + Nig increased MitoSOX Red fluorescence, dexamethasone treatment significantly inhibited MitoSOX Red fluorescence)

mtROS is critical for NLRP3‐inflammasome activation. To detect the effect of corticosteroid on mtROS, we used MitoSOX to label mtROS. Our findings of flow cytometry revealed that dexamethasone suppressed mtROS generation in macrophages stimulated with LPS and nigericin (Figure 5A). The mean fluorescence intensity of mtROS was also significantly inhibited after corticosteroid intervention (Figure 5B). Confocal microscopy showed that MitoSOX Red fluorescence increased after stimulated with LPS and nigericin, and decreased after dexamethasone treatment (Figure 5C), implying corticosteroid had a direct role on mtROS production to suppress NLRP3‐inflammasome activation.

## 4 DISCUSSION

Corticosteroids have been considered as a potential therapy for ALI/ARDS due to their anti‐inflammatory and immunomodulatory effects. However, it remains controversial and the underlying mechanism remains indefinite.^21^, ^22^ It promoted us to further explore the specific mechanisms in vivo and in vitro. Our results of animal experiment were consistent with previous studies that implicated NLRP3‐inflammasome activation and the maturation and release of IL‐1β were critical in the development of experimental ALI/ARDS.^14^, ^15^, ^17^, ^18^, ^19^ When the NLRP3‐inflammasome was knocked out, the reductions of destruction of the alveolar structure and inflammatory cell infiltrations of lung tissue, especially the number of neutrophils infiltration in BALF were significantly. Meanwhile, the concentration of inflammatory cytokines was light in serum and BALF of NLRP3‐inflammasome deficiency mice. These demonstrated that inflammatory cytokines IL‐1β and IL‐18 maturation dependent NLRP3‐inflammasome activation were essential for LPS‐induced ALI. Further, we used corticosteroid treatment for LPS‐induced ALI. Interestingly, we found dexamethasone obviously attenuated inflammatory cell infiltration in lung tissue, alveolar leakage and IL‐1β, IL‐18 release in serum and BALF. These suggested that corticosteroids played an important role on suppressing the NLRP3‐inflammasome regulated cytokines (ie, IL‐1β and IL‐18) production during the processing of ALI. We were eager to know the mechanism behind these observations.

In *vitro* models, our results presented that caspase‐1 cleavage, IL‐1β and IL‐18 secretion were almost absent in NLRP3‐deficiency macrophages during the process of acute inflammation. Moreover, when NLRP3‐inflammasome was knocked out, cell death was significantly reduced by detecting LDH in the supernatant (Figure 2). It mean that NLRP3‐inflammasome activation played an important role in acute cell injury. Thus, we speculated whether corticosteroid interfered with acute cell injury via mediating NLRP3‐inflammasome activation.

**FIGURE 6:**
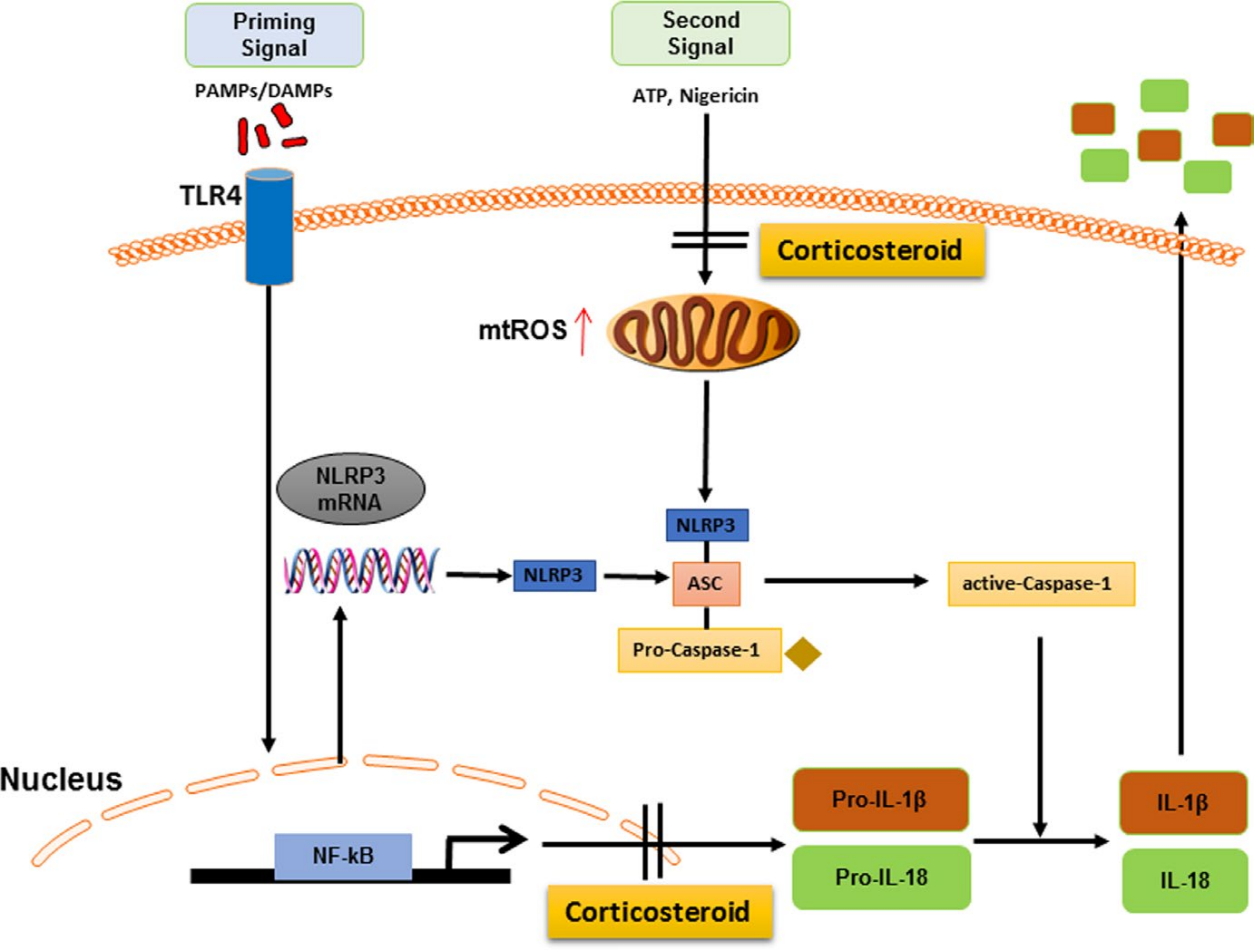
Summary of corticosteroid mediating the NLRP3‐inflammasome activation

As far as we know, for full activation of NLRP3‐inflammasome, it requires a priming signal for the production of pro‐IL‐1β‐ and pro‐IL‐18‐dependent TLR4/NF‐κB (Signal 1) and then Signal 2 was triggered by some cellular danger signals, such as ATP, nigericin or other stimuli produced the active Caspase‐1, which cleaves the pro‐IL‐1β and pro‐IL‐18 to their biologically active inflammatory factors IL‐1β and IL‐18, leading to acute inflammation (Figure 6).^23^, ^24^ In the current study, we found that corticosteroids conferred protection against membrane damage in macrophages challenged with pro‐inflammatory stimuli. When macrophages were treated with dexamethasone prior to inducing stimuli, the activation of NF‐κB (pp65) and NLRP3 protein was obviously suppressed, which were in dose‐dependent manner. Apparently, the downstream inflammatory factors of caspase‐1 activation and IL‐1β maturation were also significantly inhibited by dexamethasone. Further, the transcription level about the mRNA expression of TNF‐α, Caspase‐1 and IL‐1β, which are the classical cytokines transcribed via the NF‐κB‐dependent signalling pathway, was also suppressed by corticosteroids in dose‐dependent manner. These findings demonstrated that dexamethasone modulated inflammatory response by blocking NF‐κB signal pathway, which was consistent with the report by Held *et al*.^25^ We reached that corticosteroids inhibited LPS‐induced‐NLRP3‐inflammasome activation by blocking NF‐κB signal pathway, which was the first signal for NLRP3‐inflammasome activation.

However, in clinical practice, when corticosteroids are used, the inflammatory response has already occurred. According to our experimental data, a plausible explanation for this paradoxical behaviour is that corticosteroid can modulate the NLRP3‐inflammasome activation other than blocking the NF‐κB signalling pathway. Further, we simulated this process in vitro. Murine peritoneal macrophages were stimulated by LPS prior and then received corticosteroid treatment before ATP or nigericin stimulated. Results revealed that the caspase‐1 activation and IL‐1β secretion in cell culture supernatants were still suppressed by dexamethasone. Moreover, dexamethasone exerted protection against cell damage during the process of acute inflammatory response by inhibiting LDH release in cell culture supernatants. With the increase of dexamethasone dose, the expression of Caspase‐1, IL‐1β and NLRP3 decreased gradually, although the inhibitory effect of dexamethasone on inflammatory response was not as strong as that dexamethasone used before inflammatory reaction. However, the expression of NF‐κB (pp65) was not affected. These results suggested that when NF‐κB was activated, corticosteroid treatment directly inhibited the NLRP3‐inflammasome activation by acting on the second signal pathway of NLRP3‐inflammasome activation.

Previous studies have shown that mtROS served as a critical role triggering the activation of the NLRP3‐inflammasome.^26^, ^27^ Several researches had reported that the excess generation of mtROS hyperactivateed immune responses, which associated to some infectious diseases, and some drugs exerted their corresponding biological effects by interfering with mtROS generation.^28^, ^29^, ^30^ In Xu's study, they reported that statin use enhanced bleomycin‐induced lung inflammation and fibrosis through a mechanism involving increased mtROS generation to enhance NLRP3‐inflammasome activation.^28^ Our previous research found that macrolides protected against Pseudomonas aeruginosa infection via suppressing mtROS generation to inhibit inflammasome activation.^29^ We hypothesized whether corticosteroid affected prognosis of ALI/ARDS via intervening mtROS production. Thus, we used special fluorescent labelled mtROS to detect it. We found that dexamethasone suppressed mtROS generation in stimulated macrophages. It was consistent with Lee's work.^30^ It could be a key mechanism for treating ALI/ARDS with corticosteroids, although the specific mechanism by which corticosteroids down‐regulate mtROS remains to be explored. The investigation of other possible and important mechanisms of corticosteroid action on NLRP3‐inflammasome activation, such as nitric oxide synthase, the glucocorticoid receptor and others may be warranted.^31^, ^32^

## 5 CONCLUSIONS

In summary, NLRP3‐inflammasome activation exerts a critical role in acute inflammatory reaction. Our study has provided an interpretation of mechanism on corticosteroids regulate NLRP3‐inflammasome activation at both the transcriptional level via blocking NF‐κB signal pathway and processing level via suppressing the mtROS generation. These findings provide a new consideration of corticosteroid administration for ALI/ARDS.

## CONFLICT OF INTEREST

The authors declare that they have no conflicts of interest.

## AUTHOR CONTRIBUTION

**Jia‐Wei Yang:** Conceptualization (equal); Data curation (equal); Formal analysis (equal); Investigation (equal); Methodology (equal); Resources (equal); Software (equal); Validation (equal); Writing‐original draft (equal); Writing‐review & editing (equal). **Bei Mao:** Data curation (equal); Formal analysis (equal); Investigation (equal); Methodology (equal); Resources (equal); Software (equal); Validation (equal); Writing‐original draft (equal); Writing‐review & editing (equal). **Ru‐Jia Tao:** Data curation (equal); Formal analysis (equal); Investigation (equal); Methodology (equal); Resources (equal); Software (equal); Validation (equal); Writing‐review & editing (equal). **Li‐Chao Fan:** Data curation (equal); Formal analysis (equal); Investigation (equal); Methodology (equal); Resources (equal); Software (equal); Validation (equal); Writing‐review & editing (equal). **Hai‐Wen Lu:** Data curation (equal); Formal analysis (equal); Investigation (equal); Methodology (equal); Resources (equal); Software (equal); Validation (equal); Writing‐review & editing (equal). **Bao‐Xue Ge:** Data curation (equal); Formal analysis (equal); Investigation (equal); Methodology (equal); Resources (equal); Software (equal); Validation (equal); Writing‐review & editing (equal). **Jin‐Fu Xu:** Conceptualization (equal); Data curation (equal); Formal analysis (equal); Funding acquisition (lead); Investigation (equal); Methodology (equal); Project administration (lead); Resources (equal); Software (equal); Supervision (lead); Validation (equal); Writing‐original draft (lead); Writing‐review & editing (lead).

## Data Availability

The data in the current study are available from the corresponding author on reasonable request.
